# Circularly polarized dual-band resonance in a miniaturized implantable antenna using combined hexagonal and rhombic patches

**DOI:** 10.1038/s41598-025-86882-3

**Published:** 2025-01-28

**Authors:** Shanmugam Kumaravel, Madurakavi Karthikeyan

**Affiliations:** Department of Communication Engineering, School of Electronics Engineering, Vellore Institute of Technology, Vellore, Tamil Nadu India

**Keywords:** Implantable antenna, Circular polarization, Dual-band antenna, Biomedical antenna, SAR, Compact antenna, ISM band, UHF band, Biomedical engineering, Engineering, Electrical and electronic engineering, Implants

## Abstract

The design and characterisation of a novel dual-band implantable antenna with compact size is presented in this research. The antenna, which is $5 \times 5 \times 0.635 {\text{mm}}^{3}$ in size and operates at two critical frequencies—0.954 GHz in the UHF band and 2.4 GHz in the ISM band—was fabricated on an RT6010 substrate. The U-shaped slot and shorting pin on the radiating element have been exploited to achieved dual-band and circular polarization. The antenna is noteworthy for achieving circular polarization with a broad axial ratio bandwidth of 24.6%, which enables strong performance throughout its operating frequencies. The proposed antennas SAR values satisfy IEEE safety standards for implantable medical devices with a gain of − 28.1 dB at 2.4 GHz and − 31.2 dB at 0.954 GHz, despite its small size. The design represents a significant advancement in the field of medical implant technology since it prioritizes effective wireless communication capabilities while upholding strict safety regulations.

## Introduction

Recently, implantable antennas became vital for real-time remote health monitoring and improved device management in medical implants. They make wireless communication easy to use, which lessens the need for intrusive treatments and improves patient comfort. These antennas also enable the Internet of Things to be integrated with devices, enabling thorough health tracking and prompt actions. Furthermore, medical device functionality and compactness are enhanced by developments in antenna technology^1^. The implantable can be used for various biomedical application such as, body- centric communication^2^, continuous track of blood sugar level^3^, brain implantable technologies^4^, capsule endoscopy^5^, mobile medical device^6,7^, retinal prostheses^8^, intracranial pressure monitor^9^, and cardiac beat monitoring^10^.

The implantable antenna design become challenging, since it has many constraints^11,12^,when compare to the conventional antennas used in wearable^13,14^, traditional wireless transmission^15^. Specific design constraints such as, safety, reliable, compact size, challenges the design process^16^, which is not case in conventional antennas^17^. The federal communication commission (FCC) has approved for utilization of several frequency bands for medical purpose including, medical device radio communication service (Med Radio) (401–406 MHz)^18^, medical implantable communication services (MICS) (402–405 MHz)^19^, industrial medical device (ISM) (433–434.8 MHz; 902–928 MHz; 2.4 -2.48 GHz)^20,21^, ultrahigh frequency (UHF) (951–956 MHz)^22,23^, and wireless medical telemetry system (WMTS) (608–614 MHz; 1395–1400 MHz)^24^. RFID technology is also suitable of utilizing UHF frequency bands. Ultrahigh frequency bands (UHF) have been suggested to be used for in-body wireless communication, in addition, the UHF band can be used in RFID applications to read, track and monitor the patient^22,23^ , Industrial scientific and medical (ISM) band is used for sending a wake-up signal and wireless charging for peacemaker^30^.

Many linear polarization (LP) and circular polarization (CP) antennas has been reported in this direction^25–27^.The constant movement of the human body will affect the location of the linear polarization antenna (LP) and create the polarization mismatch and it will reduce the antenna performance. To resolve this problem, circular polarization (CP) antenna have been suggested to design. In^28^, single ISM band CP antenna was proposed with the dimension of $9.8\times 9.8\times 1.27 {\text{mm}}^{3}$. However, the size of the antenna was high. In^29^, four L-shaped opening on patch and plus shaped slot on the ground plane has resulted in achieving the CP properties at 2.4 GHz resonance band. However, low gain and high SAR restrict it practical implementation. The antenna with dual band resonance exhibiting CP property at 1.4 GHz band with axial ratio bandwidth of 10.38% and LP property at 2.4 GHz band has been reported in^30^. The antenna reported with low axial ratio bandwidth and there is a room improvement in axial ratio bandwidth. The truncated edges were used to achieve the CP performance in^31^. However, the gain of the antenna was low. The ladder-shaped structure on the radiating element improved the impedance matching and the flower petals shaped slot on ground plan achieved CP performance with an axial ratio < 3 dB at 403 MHz band in^32^. In^33^, the designed implantable antenna resonates at 2.4 GHz (ISM band) with dimension of $9.8\times 9.8\times 0.88 {\text{mm}}^{3}$ with an axial ratio bandwidth (ARBW) of 28.7%. However, the SAR value and size of the antenna was high. Using X-shaped open slot on both the patch and ground plane the CP property at 0.915 GHz and 2.45 GHz with narrow axial ratio band width has been reported in^34^. However, the volume and SAR value of the designed antenna was high.

In this work, a compact miniaturized dual-band implantable antenna resonating at 0.954 GHz and 2.4 GHz (UHF, ISM bands), with wide axial ratio bandwidth at ISM band for biomedical application, with a compact dimension of $5 \times 5 \times 0.635 {mm}^{3}$. The shorting pin is helped to achieve the miniaturization and dual-band resonance in designed antenna. The U-shaped defective ground structure leads to perfect impedance matching at the desired frequency bands (UHF and ISM) and satisfy the circular polarization property at 2.4 GHz. The proposed antenna exhibits the dual-band resonance at 0.954 GHz with linear polarization (LP) property and circular polarization (CP) property at 2.4 GHz. The realized peak gain of the proposed antenna was -31.2 dBi and -28.1dBi at 0.954 GHz and 2.4 GHz resonance, respectively.

The designed antenna was placed at 4 mm depth from the top of the homogenous skin phantom with the dimension of $100\times 100\times 50 {\text{mm}}^{3}$ in CST simulation environment. The proposed antenna was fabricated and tested on skin-mimicking gel and minced pork meat in order to verify the simulation results.

The remaining portions of this work are organized as follows. Section "Design of proposed antenna" presents the design of proposed antenna, evolution stages and parametric study. Simulated and measured results are discussed in section "Simulated and measurement results and discussion". Finally, a conclusion is given in section "Conclusion".

## Design of proposed antenna

### Geometry view of proposed antenna

**Fig. 1:**
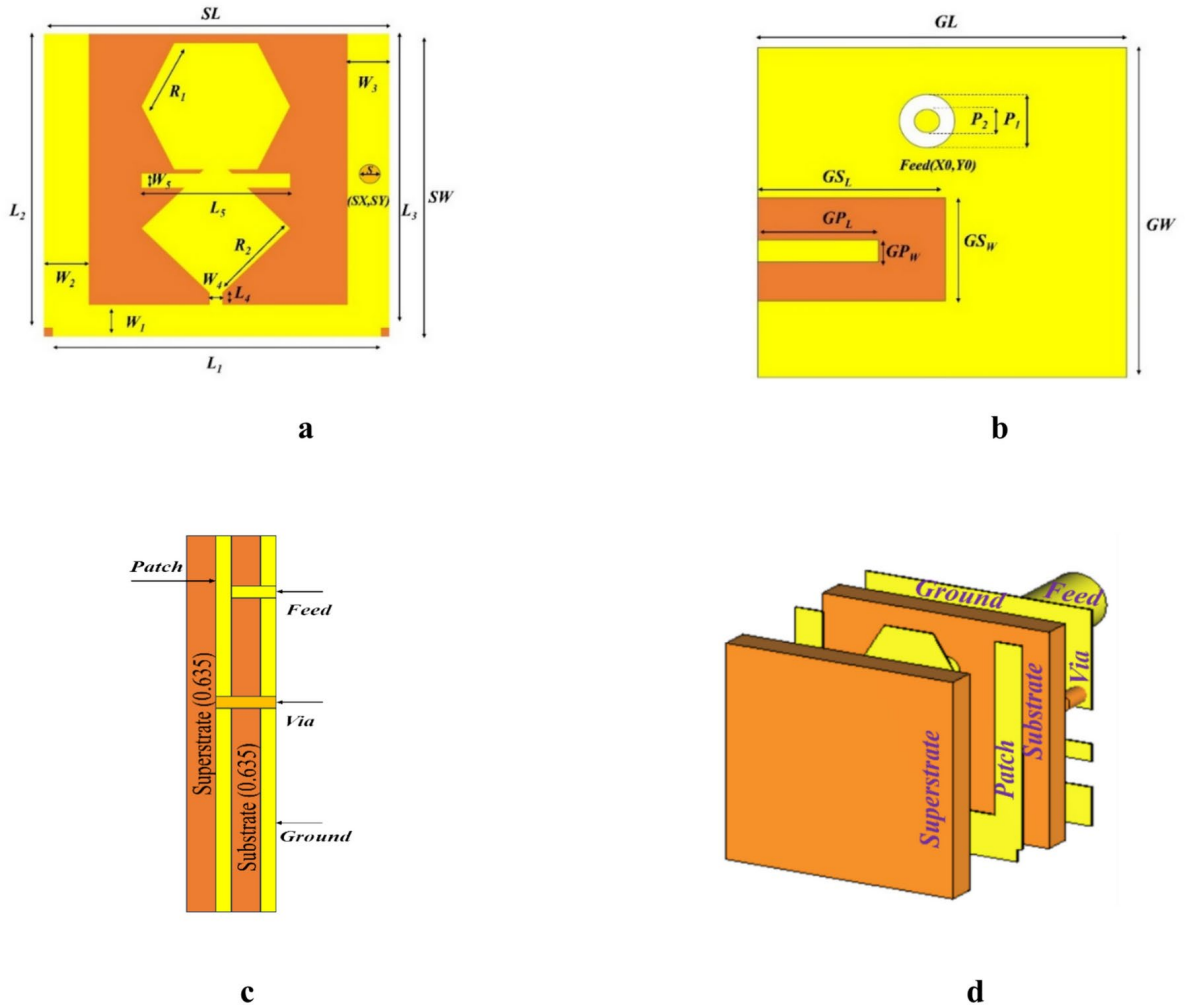
Geometry of proposed antenna: (**a**) Front, (**b**) back, (**c**) side, and (**d**) Isometric view.

The geometry and dimension of the proposed antenna are shown in Fig. 1. The hexagonal and rhombic shaped patch act as radiating element is shown in Fig. 1a. The U-shaped slot in the ground plane used to achieve the miniaturization of the antenna is shown in Fig. 1b. The side and isometric view of the proposed antenna are show in Fig. 1c and d, respectively. We used high dielectric biocompatible material RT/Rogers 6010 (ε_r_ = 10.2, tan δ = 0.0023) for both substrate and superstrate with a thickness of 0.635 $mm$. Superstrate is used to avoid the direct contact between human tissues and the antenna. In order to achieve the miniaturization and desired resonance frequency band, a high dielectric material was chosen. The overall dimension of the proposed antenna is $5\times 5\times 1.27 {\text{mm}}^{3}$. The dimension of the proposed antenna in terms of λ_0_ is 0.0159 λ_0_ × 0.0519 λ_0_ × 0.004 λ_0_. Table 1 represent the detailed parameter list of the designed antenna.

**Table 1:** Various parameter values of proposed antenna.

Geometry parameter	Dimension (mm)	Geometry parameter	Dimension (mm)
L_1_	4.8	W_1_	1
L_2_, L_3_	4.7	W_2_, W_3_	0.6
L_4_	0.3	W_4_	0.35
L_5_	2	W5, S	0.2
R_1_	1.1	R_2_	1.1
SL, GL	5	SW, GW	5
(X0, Y0)	(0.25, 1.25)	P_1_	0.6
(SX, SY)	(2.2, 0.5)	P_2_	0.3
GS_L_	2.7	GP_L_	0.6
GS_W_	1.8	GP_W_	0.4

### Evolution stages of proposed antenna

**Fig. 2:**
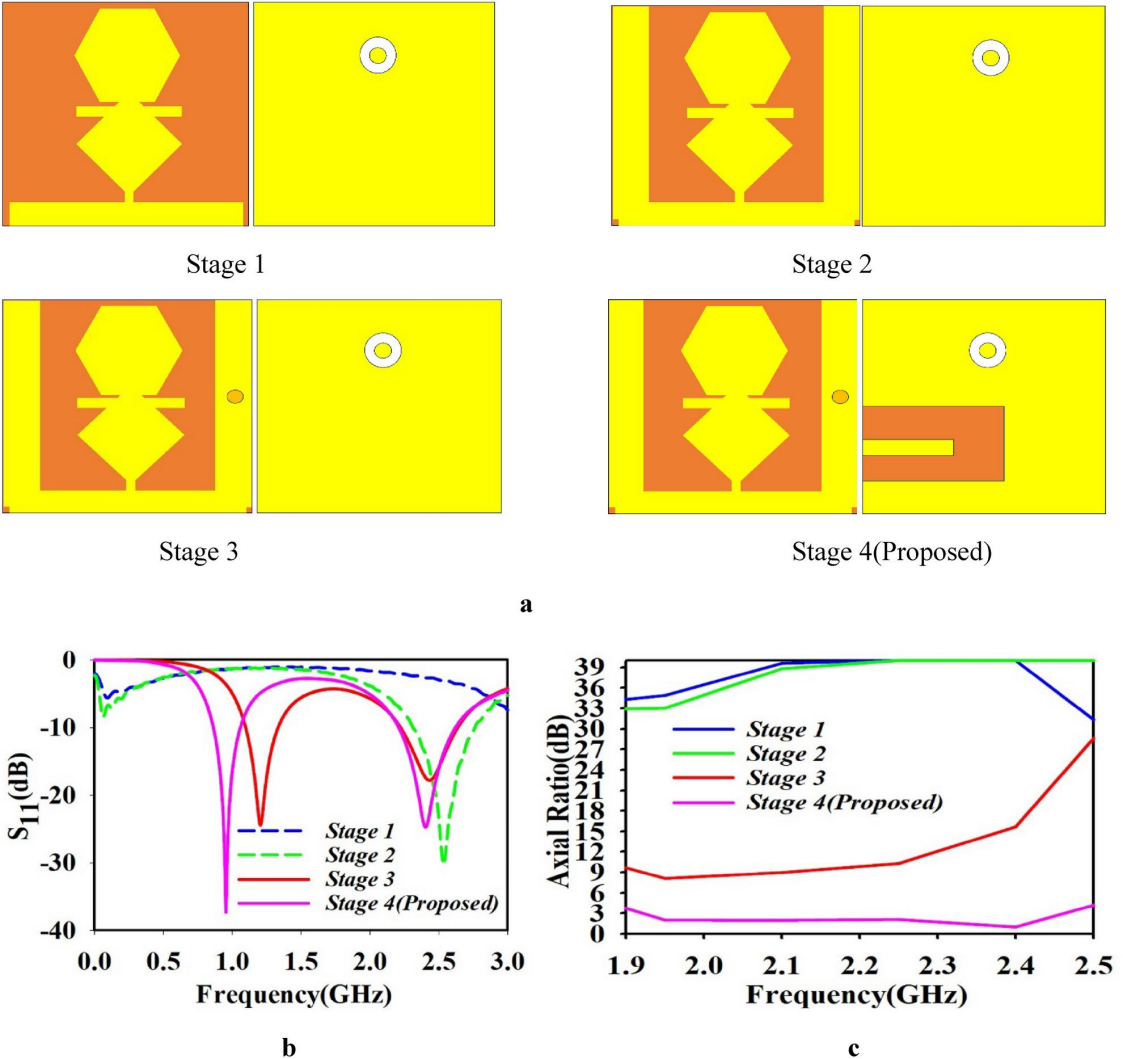
(**a**) Evolution stages of the designed antenna, (**b**) Reflection coefficient (S_11_) comparison for various evolution stages, and (**c**) axial ratio comparison of the various evolution stages.

The suggested miniaturized and circularly polarization antenna undergoes four stages of optimization during evolution, as shown in Fig. 2a. The corresponding reflection coefficient S_11_, and axial ratio performance of different stages are shown in Fig. 2b and c, respectively. Initially the proposed antenna was initiated with hexagonal and rhombic shaped patch on a Rogers RT/duroid 6010 substrate with the dimension of $5\times 5\times 0.635 {\text{mm}}^{3}$ with full ground structure as shown in Fig. 2a. At this stage, the resonance was not achieved for any medical implantable application and corresponding axial ratio was greater than 3 dB as shown in the Fig. 2b and c. Next, a pair of resonators with same length and width was added in initial design. Through this stage 2, the antenna resonates at 2.5 GHz with S_11_ < − 25 dB, as shown in Fig. 2b. However, the relevant axial ratio is still greater than 3 dB, as shown in Fig. 2c. In stage 3, a shorting pin were introduced in the right-side arm of the radiating patch at SX, SY. This resulted in stage 3 leads, to a dual-band antenna resonating at 1.2 GHz and 2.5 GHz with the return loss S_11_ of − 24.7 dB and − 17.92 dB, respectively as shown in Fig. 2b. At this stage the axial ratio was shifted to 15 dB as shown in Fig. 2c. Still, the desired frequency band and circular polarization have not been achieved. Hence, to achieve desire dual-band and circularly polarization, a U-shaped ground slot was introduced in the ground plan in stage 4. This leads to a desired dual band resonance frequency of 0.954 GHz and 2.4 GHz with return loss of − 37.31 dB and − 24.73 dB, respectively as shown in Fig. 2b. the corresponding axial ratio was achieved at less than 3 dB for the 2.4 GHz resonant frequency as shown in Fig. 2c.

### Parametric study of proposed antenna

An extensive parametric study of various important parameter on the antenna impedance matching is described in this section. The key parameter considered are rhombus radius R_2_, arm length L_2_, width W_5_, and location of the shorting pin.

#### Effect on variation in rhombic radius R_2_

**Fig. 3:**
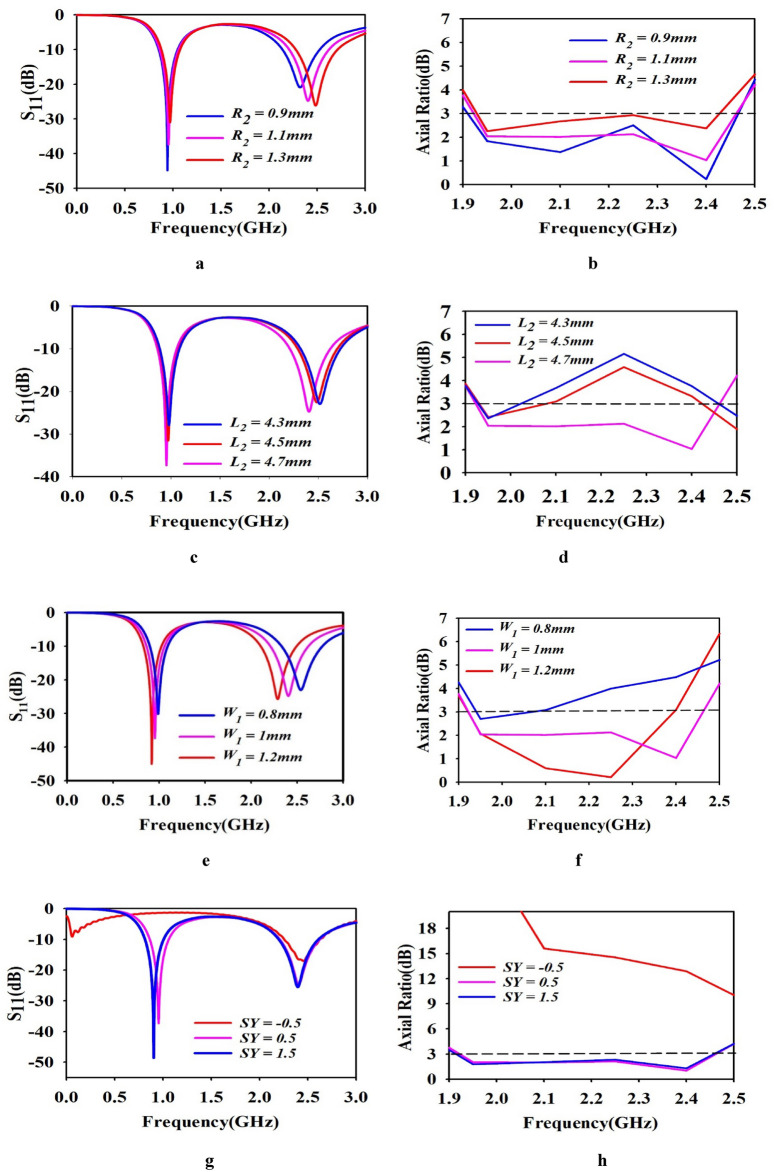
(**a**) Effect of Variation in rhombic radius R_1_ on antenna performance and (**b**) Variation of axial ratio. (**c**) Effect of Variation in Length L_1_ on antenna performance and (**d**) Variation of axial ratio (**e**) Effect of variation in width W_1_ on antenna performance and (**f**) variation of Axial ratio, (**g**) Effect of variation in shorting pin location on antenna performance and (**h**) variation of axial ratio.

Initially the designed antenna rhombus radius R_2_ was fixed at 1.1 mm. The variation of the rhombus radius from 0.9 to 1.3 mm and corresponding axial ratio as shown in Fig. 3a and b. When the rhombus radius R_2_ increase to 1.3 mm, the return loss S_11_ slightly moves towards right and corresponding axial ratio is shown in the Fig. 3a and b. When R_2_ = 0.9 mm, S_11_ moves towards right side which is not the desired frequency band, and relevant axial ratio was shown in Fig. 3a and b, respectively.

#### Effect of variation in length L_2_

We observed that the designed antenna circular polarization property largely depends on the length L_2_. Hence, we analysed the CP performance of the antenna by varying L_2_ from 4.3 to 4.7 mm in steps of 0.2 mm. We can observe from Fig. 3c and d that the return loss S_11_ is slightly moves towards right side, while axial ratio is > 3 dB, when L_2_ = 4.3 mm. While increasing L_3_ to 4.5 mm, the resonance frequency shifted to left side and corresponding axial ratio was still > 3 dB, as shown in Fig. 3c, and d, respectively. When we fixed that L_2_ = 4.7 mm, resonance frequency falls on desire band UHF (0.954 GHz) and ISM (2.4 GHz) and the axial ratio was < 3 dB at ISM band, as shown in Fig. 3c, and d, respectively.

#### Effect of variation in width W_1_

The impact of W_1_ on the antenna performance has been evaluated. The width W_1_ is a crucial element to determine the return loss S_11_ and circular polarization property of the proposed antenna. The W_1_ is varied in steps of 0.2 mm (from 0.8 to 1.2 mm). The designed antenna W_1_, was 1 mm initially. By varying W_1_ from 1 to 0.8 mm, the return loss S_11_ moves towards right side and corresponding axial ratio was > 3 dB, as shown in Fig. 3(e) and (f). By increasing W_1_ to 1.2 $mm$, the resonance frequency shifted to left side, while the axial ratio < 3 dB which is not our desired band. Next, we fixed W_1_ = 1 mm*,* this results in achieving the resonance at desired band and circular polarization at 2.4 GHz.

#### Effect of variation in shorting pin location

This section is describing impact of shorting pin location of the designed antenna. Figure 3g and h shows the variation of S_11_ and axial ratio respectively. When varying shorting pin location from SX = 2.2, SY = − 0.5, SX = 2.2, SY = 1, and SX = 2.2, SY = 1.5. By varying the SX = 2.2, SY = − 0.5 the return loss S_11_ fall on 2.5 GHz. At this stage, single band resonate is observed with less impedance matching and relevant axial ratio was greater than 3 dB. When SX = 2.2, SY = 1.5, the antenna produced the dual band frequency at 0.896 GHz and 2.38 GHz with return loss S_11_ of − 49.2 dB and − 26 dB, with axial ratio was < 3 dB. At this stage, even though the axial ratio was < 3 dB, the resonance frequency not fall on desired band. When setting SX = 2.2, SY = 0.5, the antenna resonates at desired frequency of 0.954 GHz (UHF) band and 2.4 GHz (ISM) band with return loss S_11_ of − 37.52 dB and − 25 dB and corresponding axial ratio was < 3 dB for 2.4 GHz ISM band. Hence, we fixed the shorting pin location at SX = 2.2, SY = 0.5.

### Mechanism of circular polarization and equivalent circuit model of the designed antenna

**Fig. 4:**
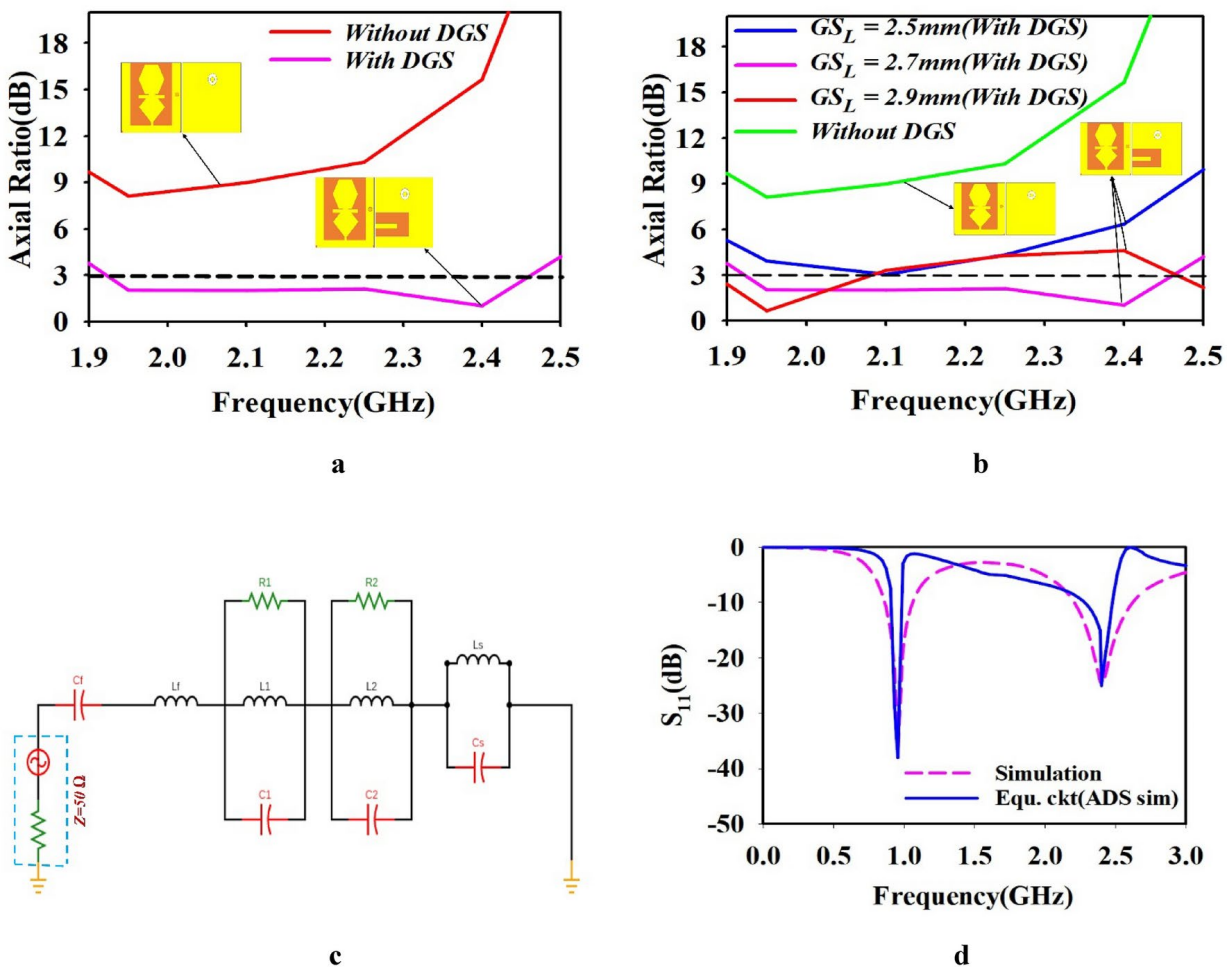
(**a**) Axial ratio with and without DGS and (**b**)Axial ratio for various value of GS_L_ (**c**) Equivalent circuit model and (**d**) Simulated S_11_-parameter in ADS.

According to the Fig. 4a, the defective ground slot (DGS) has play vital role in the circular polarization property of the designed antenna. Figure 4a, shows the axial ratio was > 3 dB without ground slot. After introduced the U-shaped slot in ground plan it exhibits circular polarization property. According to this, the analysis was made in the ground slot by varying the ground slot length GS_L_ from 2.5 to 2.9 mm. The effect of variation in ground slot length GS_L_ is addressed in the Fig. 4b. When GS_L_ is varied from 2.5 to 2.7 mm and 2.7 mm to 2.9 mm the axial ratio was shifted upward to > 3 dB at ISM band. When GS_L_ = 2.5 $mm$, the designed antenna exhibits circular polarization with optimal axial ratio of < 3 dB as shown in Fig. 4b.

### Equivalent circuit model

An equivalent circuit model of designed antenna using lumped element is shown in Fig. 4c. The Keysight advanced design system (ADS) software was used to design the equivalent circuit model of the designed antenna. A dual frequency band can be effectively described in terms of antenna resonance, as an array of RLC lumped section. The Lf and Cf values match the 50-Ω coaxial feeding line^35^ . The parallelly connected lumped element presents in left side R1, L1, and C1 depends on the hexagonal shape and central element of the radiating patch, while right side R2, L2, and C2 were represent the bottom and arm structure of the radiating patch. Moreover, the parallel Ls and Cs represent the shorting pin^32^. Both RLC tank circuit control the impedance matching of the operating frequency of the designed antenna^32,35^. Figure 4c, shows the final equivalent circuit model of the designed antenna. The comparison between simulation and equivalent circuit model results are shown in Fig. 4d. It can observe that the equivalent circuit performance matches well with simulated results. The equivalent circuit model parametric value of the proposed antenna presents in Table 2.

**Table 2:** Parametric value of equivalent circuit model (Units: Ω, nH, pF).

Symbol	Lf	Cf	R1	L1	C1	R2	L2	C2	Ls	Cs
Value	1.199	1.03	373.156	2.7502	8.9686	8.9686	6.26	1.3866	0.5	8.13

## Simulated and measurement results and discussion

**Fig. 5:**
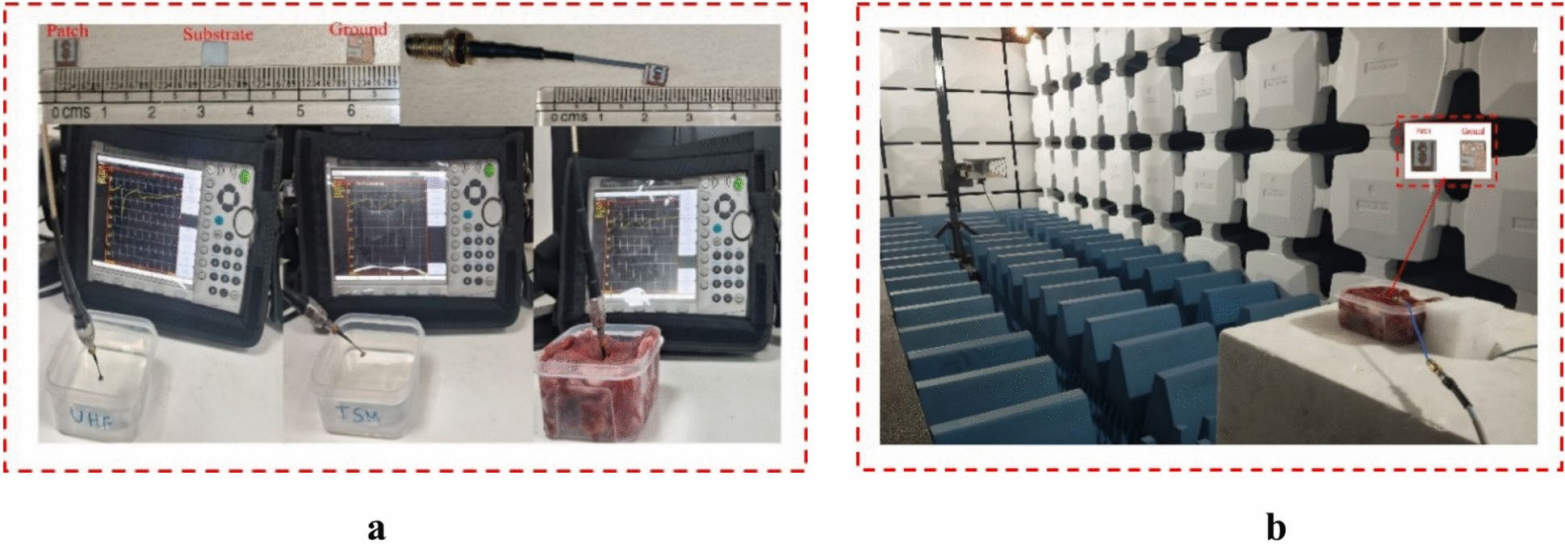
Fabricated antenna measurement and setup (**a**) Measurement setup for reflection coefficient S_11_ in saline solution and minced pork. (**b**) Measurement setup in anechoic chamber to measure the far-field radiation Pattern.

To verify the outcomes of the simulation of designed antenna, the antenna was fabricated and measured using Anritsu MS2027C vector network analyser, which operate from 5 kHz to 15 GHz frequency range. The measurement setup is shown in Fig. 5a. To validate the performance of the fabricate antenna, the antenna measured using saline solution which is performed to mimicking the skin phantom^22,30^ and minced pork meat for both UHF and ISM bands. Figure 5b shows the anechoic chamber measurement setup to measure the far-field radiation pattern of the fabricated antenna.

**Fig. 6:**
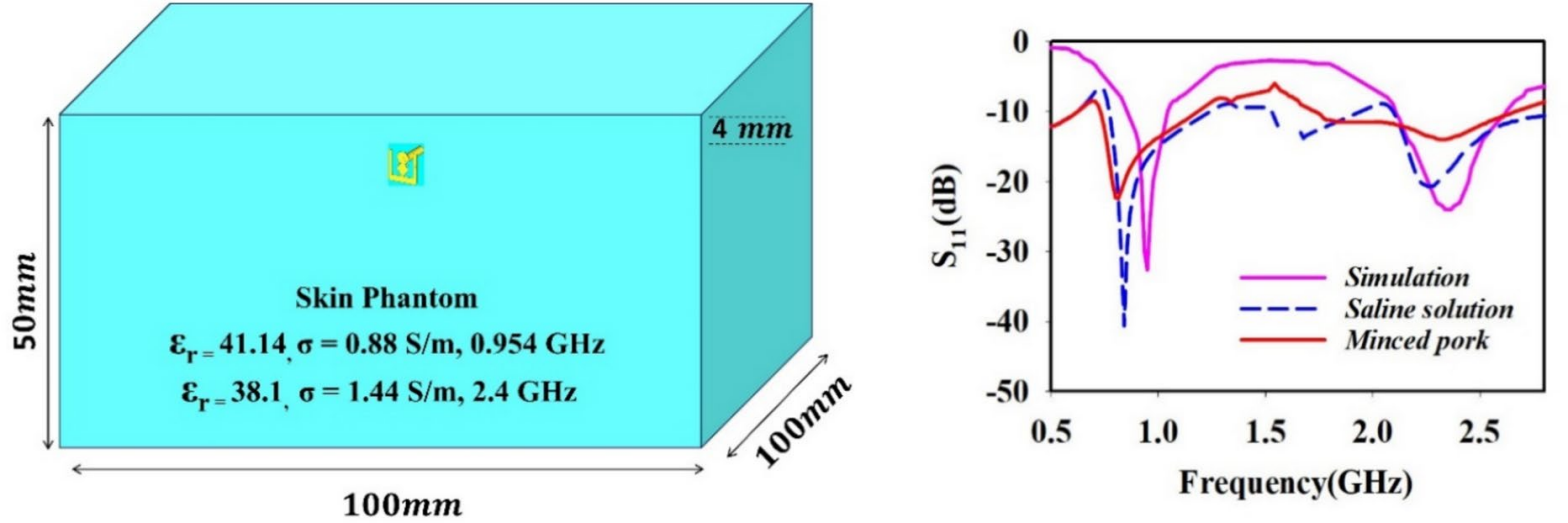
(**a**) Simulation environment of antenna in homogeneous skin phantom (**b**) Simulation and measured reflection coefficient S_11_ of the proposed antenna.

Figure 6a shows the simulation environment of homogenous skin phantom with dimension of 100 × 100 × 50 mm^3^. The antenna was placed at 4 mm depth from top of the skin. For simulation, the Bio tissues/Skin’s dielectric permittivity ε_r_ = 41.41 and loss tangent δ = 0.88 S/m at 0.954 GHz^22^ and permittivity ε_r_ = 38.1 and loss tangent δ = 1.44 S/m at 2.4 GHz^30^, respectively has been considered. The reflection coefficient S_11_ of designed antenna measurement was carried out using skin mimicking gel and minced pork. The simulation and measurement results are well matched as shown in Fig. 6b.

**Fig. 7:**
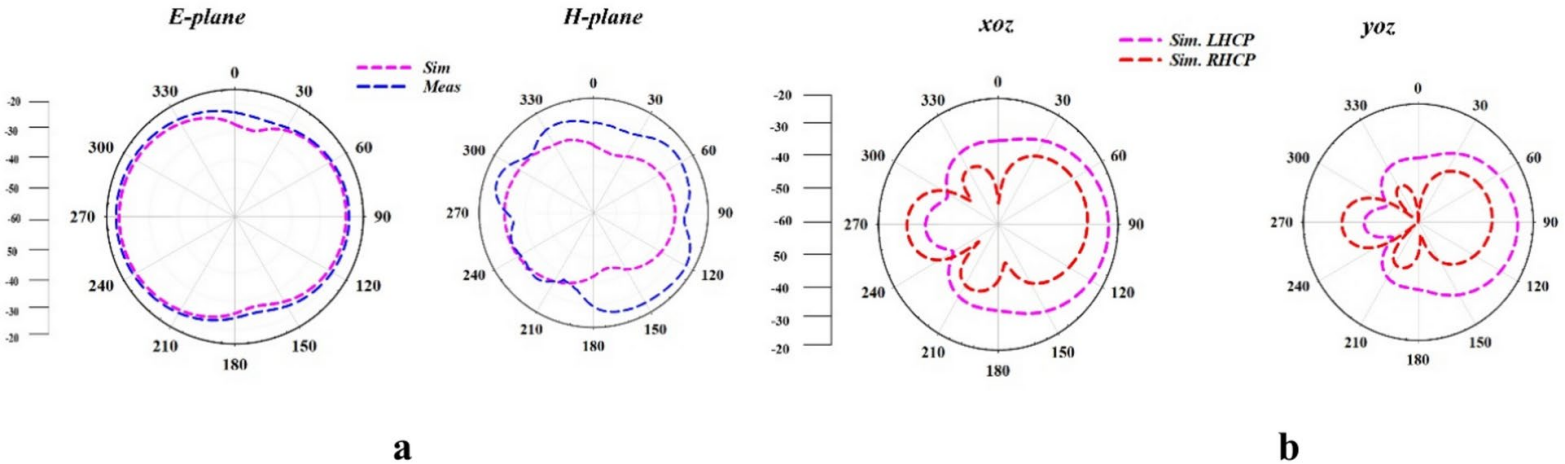
(**a**) E & H far-field radiation pattern of the designed antenna at 0.952 GHz and (**b**) *xoz* & *yoz* radiation pattern of the designed antenna at 2.4 GHz.

The far-field radiation pattern of designed antenna at 0.954 GHz and 2.4 GHz are presented in Fig. 7(a & b). The designed antenna exhibits the linear polarization at 0.954 GHz UHF band and circular polarization at 2.4 GHz ISM band. The E- and H- field radiation pattern at 0.954 GHz is shown in Fig. 7a. In the E-plane, the simulated and measured results show good agreement with minor variations in the back-lobe region. Due to external factors influencing the antenna’s performance, measurements typically show a wider beamwidth in the measured pattern than in the simulated pattern. There is a greater difference between the measured and simulated findings in the H-plane. There are some variations in the general shape of the detected radiation pattern, especially when the coverage is wider. This could be attributed to the dielectric properties of the surrounding tissues, as implantable antennas are highly sensitive to their environment. The *xoz* and *yoz* radiation field pattern at 2.4 GHz are shown in Fig. 7b. The radiation pattern in both of the plane clearly separates the circular polarizations on the left and right hands. While the RHCP exhibits reduced amplitude, indicating significant polarization selectivity, the LHCP dominates across most angles and displays consistent radiation behaviour. The simulated radiation patterns confirm that the antenna achieves effective circular polarization, predominantly favouring left-hand circular polarization at 2.4 GHz. This polarization characteristic enhances the robustness of wireless communication for implantable medical applications, as it ensures better signal penetration through the human body and reduces signal degradation caused by multipath effects. The maximum realized peak gain of designed antenna in simulation environment for UHF and ISM bands are -31.2 dBi and -28.1 dBi, respectively.

**Fig. 8:**
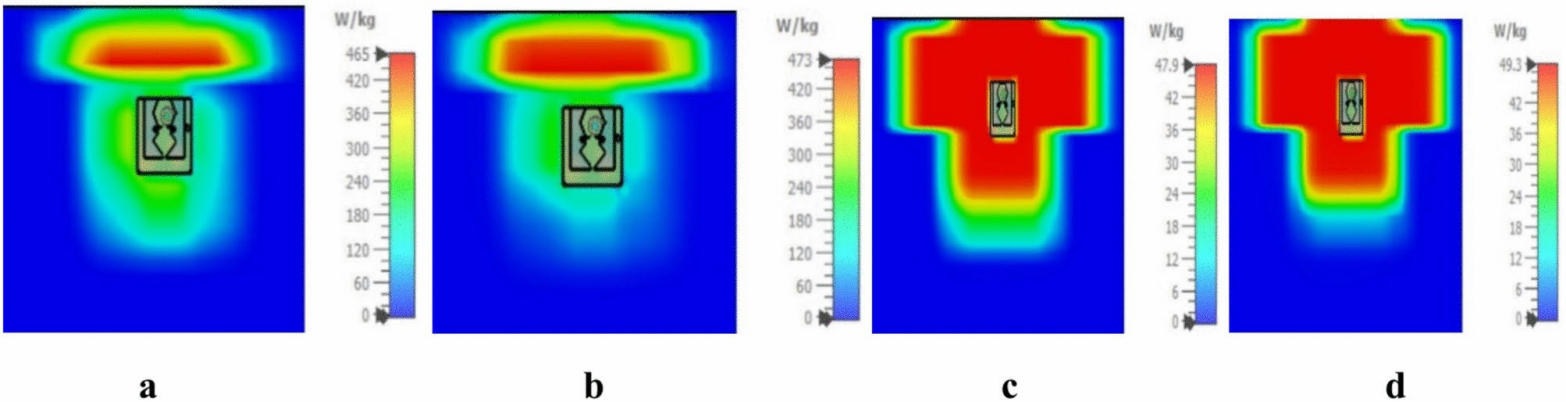
Simulated SAR (**a**) at 0.954 GHz 1 g tissues model, (**b**) at 2.4 GHz 1 g tissues model, (**c**) at 0.954 GHz 10 g tissues model, and (**d**) at 2.4 GHz 10 g tissues model.

Average SAR is the crucial value in implantable antenna which ensure patient safety. Hence, we evaluated SAR at 0.954 GHz and 2.4 GHz over 1W input power. The SAR values are tabulated in Table 3. According to the IEEE C95.1-1999 standards, the maximum SAR value over any 1 g tissue should be less than 1.6 W/kg^36^ and IEEE 95.1-2005 standards, the average SAR value over any 10 g of tissue should be less than 2 W/kg^37^. Figure 8a and b shows that simulated SAR value over 1 g tissue model was 465 W/kg and 473 W/kg, respectively. This constrains the allowable average input power should be less than to 3.44 mW and 3.88 mW, respectively. From the Fig. 8c and d the simulated SAR value over 10 g tissue model was 47.9 W/kg and 49.3 W/kg, respectively. Hence, the maximum permissible input power is 41.75 mW and 40.56 mW for UHF and ISM bands, respectively.

**Table 3:** Simulated SAR values (input power = 1 W) and computed maximum input power at dual frequency.

Tissue	Resonant frequency (GHz)	Avg. SAR(W/kg)		Max. input power (mW)	
		1 g model	10 g model	1 g model C95.1–1999 (mW)	10 g model C95.1–2005 (mW)
Skin	0.954	465	47.9	3.44	41.75
	2.4	473	49.3	3.38	40.56

**Fig. 9:**
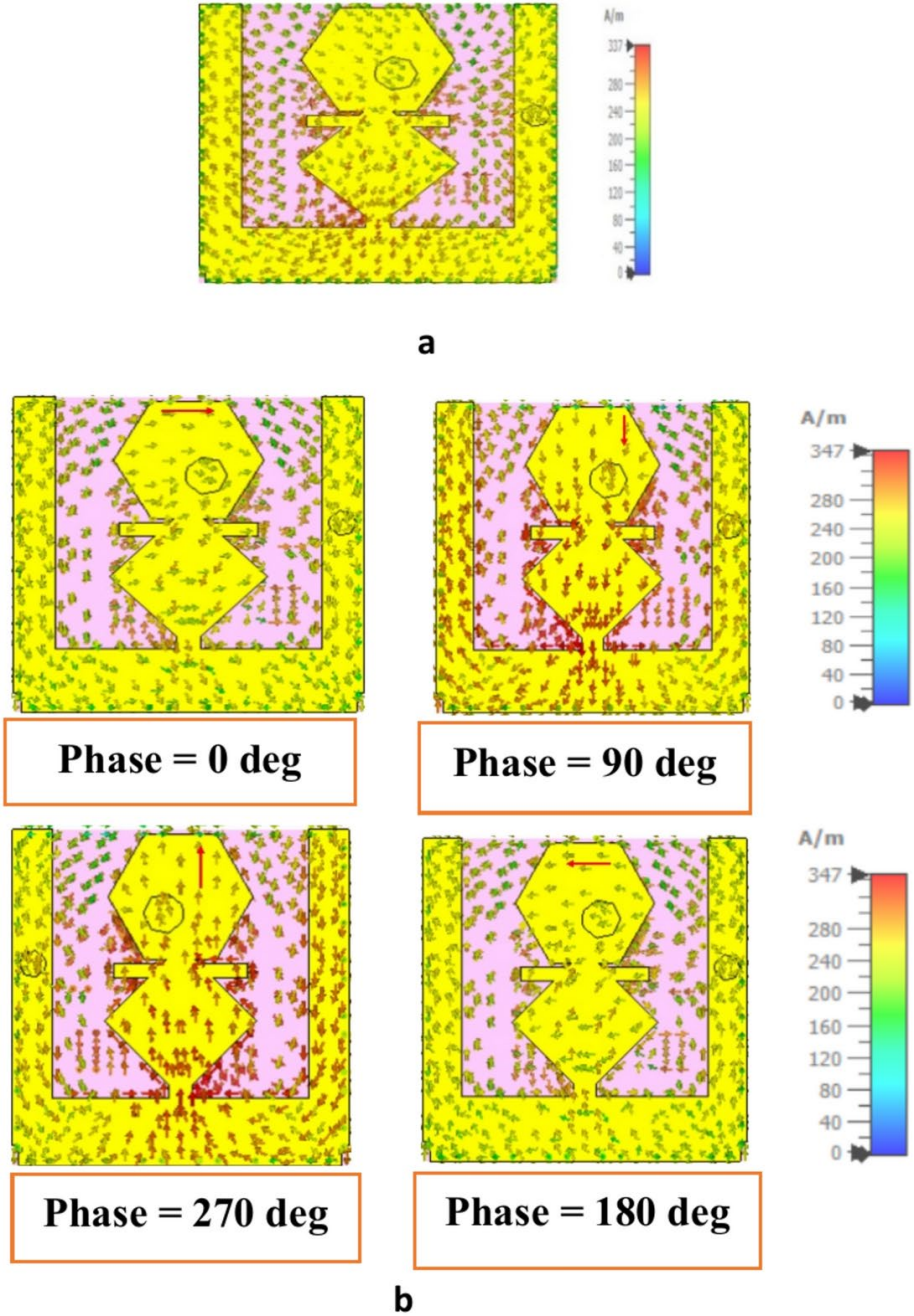
Simulated surface current distribution of proposed antenna (**a**) at 0.954 GHz and (**b**) at 2.4 GHz for four different phase excitations.

To understand polarization of the designed antenna at UHF and ISM bands, the surface current distribution is simulated at resonance frequency. The most of the current flows in the bottom of the radiating path, nearby shorting, and rhombic shaped structure at 0.954 GHz as shown in Fig. 9a. In Fig. 9b, the current distribution at four phase excitations (0°, 90°, 180°, and 270°) at 2.4 GHz is shown. The combined surface current travels to two orthogonal direction and surface current rotates the clockwise direction over a certain time period, thus the antenna exhibits left-handed circularly polarization (LHCP).

Table 4 represent the comparison of the proposed antenna with latest implantable antennas. In comparison table the designs reported in^28,29,31,32^ and^33^ have only reported single band resonance with high volume and high SAR value when compared with our designed antenna. Even though^30^, and^34^, resonates at dual-bands, the proposed antenna achieves dual-band and circular polarization with less volume. Additionally, our antenna achieves wide axial ratio bandwidth and low SAR value when compare to existing antennas.

**Table 4:** Comparison of the proposed implantable antenna with recent works.

Ref	Frequency	Size mm^3^	Volume *mm*^3^	Single/Dual band	Gain (dBi)	ARBW (< 3 dB)	1-g SAR (W/K g)	10-g SAR (W/Kg)
^28^	2.4	$9.8 \times 9.8 \times 1.27$	121.97	Single	− 33	15.8	486	90
^29^	2.4	$\pi \times {4}^{2}\times 1.27$	63.80	Single	− 37.36	2.16–2.70	856.45	92.5
^30^	1.4	$\pi \times {5.1}^{2}\times 1.27$	103.7	Dual	− 32	10.38	692	85.6
	2.45				− 31.6	–	786.9	90.9
^31^	2.4	$7.5 \times 7.5 \times 1.27$	71.4	Single	− 32.8	16.9	–	71.5
^32^	403	$9.5 \times 9.5 \times 0.64$	57.6	Single	− 31.2	30.5	229	–
^33^	2.4	$9.8\times 9.8 \times 0.889$	85.37	Single	− 24.7	28.7	697.5	89.4
^34^	0.915	$\pi \times {4.7}^{2}\times 0.889$	61.66	Dual	− 29.5	22.4	585.1	–
	2.45				− 19.2	9.48	462.2	–
This work	0.954	$5 \times 5 \times 1.27$	31.75	Dual	− 31.2	–	465	47.9
	2.4				− 28.1	24.6	473	49.3

### Link margin analysis for wireless communication

To understand the reliability of transmission to an external device, a link budget calculation was carried out at three distinct date rate (Br) 10 Kbps, 100 Kbps and 1 Mbps at 0.954 GHz and 2.4 GHz operating frequency with transmitting gain (Gt) of − 31.2 dB and − 28.1 dBi. According the European research council regulation, the input transmitter power (Pt) was fixed at -46.02dBW (25 μW)^9^. At both the frequency of the implantable antenna 2.15 dBi was considered as constant gain (Gr) of the external dipole antenna. The power difference between the desired and available levels as determined by the link margin (LM), is

1 $$Lm\left(dB\right)=\left(Pa-\text{Pr}\right),$$

2 $$Pa\left(dB\right)=Pt+Gt+Gr-Lf-N0),$$

3 $$\text{Pr}\left(dB\right)=\frac{Eb}{N0}+10log10\left(Br\right)-Gc-Gd,$$

4 $$Lf={\left(\frac{4\pi d}{\lambda }\right)}^{2}$$

**Fig. 10:**
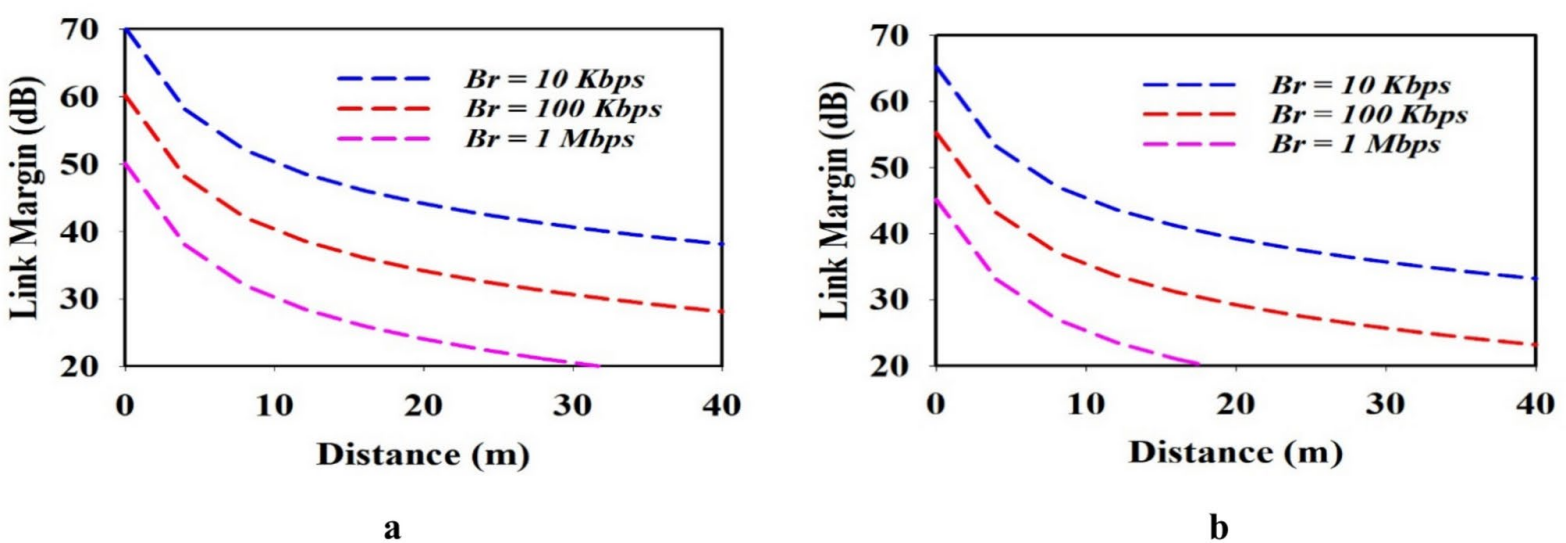
Link margin for different data rates (**a**) at 0.954 GHz and (**b**) at 2.4 GHz.

For a reliable communication, a 20 dB link margin is taken into consideration. In both operating frequency bands, communication cloud be achieved at a distance greater than 30 and 15 m at 0.954 GHz and 2.4 GHz, respectively as clearly shown in Fig. 10a and b Therefore, the proposed implantable antenna is suitable for data telemetry application.

## Conclusion

In this proposed work, the compact miniaturized antenna was designed to produced dual-band operation for biotelemetry application. The dimension of the designed antenna was $5 \times 5 \times 0.635{\text{mm}}^{3}$ with LP/CP properties. The dual-band resonance and circular polarization mainly achieving due to the combination of shorting pin and U-shaped slotted ground. This combination helps in achieving wide axial ratio bandwidth of 24.6%. To validate the simulation results, the designed antenna was fabricated and measured by using minced pork meat and skin-mimicking gel. The measured results are well matched with simulation results. The suggested antenna complies with IEEE C 95.1-1999 and IEEE C 95.1-2019 safety standards at permissible input power levels. The presented antenna is a desired option for implantable medical systems due to compact miniaturized size, dual band resonance and circularly polarization with wide axial ratio bandwidth.

## Data Availability

All data generated or analysed during this study are included in this article.
